# Bioactive Nanoemulsions for Enhancing Sausage and Meat Patty Shelf-Life

**DOI:** 10.3390/foods15030430

**Published:** 2026-01-24

**Authors:** Antia G. Pereira, Ana Perez-Vazquez, Paula Barciela, Ana O. S. Jorge, Ezgi Nur Yuksek, Rafael Nogueira-Marques, Sepidar Seyyedi-Mansour, Miguel A. Prieto

**Affiliations:** 1 Instituto de Agroecoloxía e Alimentación (IAA), Universidade de Vigo, Nutrition and Food Group (NuFoG), Campus Auga, 32004 Ourense, Spain; antia.gonzalez.pereira@uvigo.es (A.G.P.); ana.perez.vazquez@uvigo.es (A.P.-V.); paula.barciela@uvigo.es (P.B.); anolijorge@gmail.com (A.O.S.J.); ezginur.yuksek@uvigo.gal (E.N.Y.); nogueirarafael29@gmail.com (R.N.-M.); sepidar.seyyedi@uvigo.es (S.S.-M.); 2 Galicia Sur Health Research Institute (IIS Galicia Sur), Nutrition and Food Group (NuFoG), SERGAS-UVIGO, 36213 Vigo, Spain; 3 REQUIMTE/LAQV, Department of Chemical Sciences, Faculty of Pharmacy, University of Porto, R. Jorge Viterbo Ferreira 228, 4050-313 Porto, Portugal

**Keywords:** nanoemulsions, bioactive compounds, shelf-life extension, natural preservers, meat patties, sausages

## Abstract

The application of bioactive nanoemulsions in the meat industry has attracted great interest due to their ability to improve the stability, bioavailability, and functionality of bioactive compounds, contributing to the extension of the shelf-life of highly perishable products, such as sausages and meat patties. Thus, this review provides a critical analysis of the application of nanoemulsions in sausages and meat patties, with emphasis on their mechanisms of action, formulation strategies, and performance in improving oxidative stability and microbial safety. Nanoemulsions, typically characterized by droplet sizes below 200 nm, increase interfacial area and penetration into meat matrices, resulting in reductions of 30–60% in lipid oxidation markers and decreases of 1–2 log CFU/g in spoilage and pathogenic microorganisms. Preparation and stabilization approaches, including high-energy and low-energy methods, are summarized, and the influence of nanoemulsion characteristics on texture, color, pH, and sensory perception is discussed. Particular attention is given to technological barriers, such as scale-up feasibility, stability during processing and storage, interactions with meat proteins, as well as regulatory and labeling considerations related to nano-enabled foods. Overall, the current evidence indicates that NEs represent a viable strategy to replace synthetic preservatives while supporting clean-label product development; however, further research on safety assessment, optimal dosing, and consumer acceptance is still required for broader industrial implementation.

## 1. Introduction

Feeding the world’s population in a sustainable, equitable, nutritious, and economically efficient manner represents a major global challenge, for which technological innovation has been proposed as a partial solution [1]. Although evidence indicates that meat consumption has stabilized in several countries, intake continues to rise in many emerging markets, driven primarily by increased affordability [2]. In fact, the available data indicate that economic development and urbanization have contributed to a substantial expansion in global animal protein consumption over the past five decades. This trajectory is anticipated to continue, with an additional 14% increase projected by 2030 [3,4].

Meat is generally classified according to its source and degree of processing. Broadly, it can be divided into red meat, poultry, their respective processed meat products, and other minor categories, such as lamb or game [5]. Among these meat categories, the most widely produced is poultry, with a noticeable decrease in red meat [3]. Processed meat typically includes products such as sausages, ham, bacon, cured or smoked meats, and ready-to-eat meats, reaching an estimated 228 million tons in 2024. Among processed-meat types, “prepared or preserved meats” (e.g., canned, cured, or shelf-stable products) accounted for about 145 M tons, representing approximately 63% of total processed-meat volume, followed by sausages and similar products (~48 M tons), then by salted, dried, or smoked meats plus offal (~25 M tons) [5,6].

Regarding their nutritional composition, these products are typically characterized as a relevant source of B-complex vitamins, such as thiamin (B1), riboflavin (B2), niacin (B3), pantothenic acid (B5), vitamin B6, biotin (B7), folacin/folate (B9), and vitamin B12. Furthermore, they are excellent sources of several minerals, such as iron (Fe), copper (Cu), zinc (Zn), and manganese (Mn), and play an important role in the prevention of Zn and Fe deficiencies [7]. However, despite their appealing nutritional profile, processed-meat product commercialization is associated with notable quality, safety, and preservation challenges. For instance, their elevated fat content increases susceptibility to lipid oxidation, while high moisture levels can promote microbial growth, and the use of curing agents or additives may lead to the formation of undesirable chemical compounds during processing or storage [8]. Together, these factors contribute to increased economic losses in the meat industry, in which it is estimated that up to 23% of the production is lost and wasted [9]. These loses are particularly noticeable in emulsion-type meat products, such as meat patties and sausages, products with 20–30% of fat content incorporated as an emulsion with a prominent level of saturated fatty acids (SFAs) [10]. Thus, shelf-life limitations, together with growing concerns about environmental impact, have prompted a rising consumer demand for sustainable and natural additives that can maintain product quality while minimizing potential health risks [10]. As a result, numerous studies have been conducted to evaluate the use of natural additives in processed meat products [11]. However, the evaluation of the incorporation of bioactive NEs, specifically in sausages and meat patties, to extend their shelf-life is still limited.

**Figure 1:**
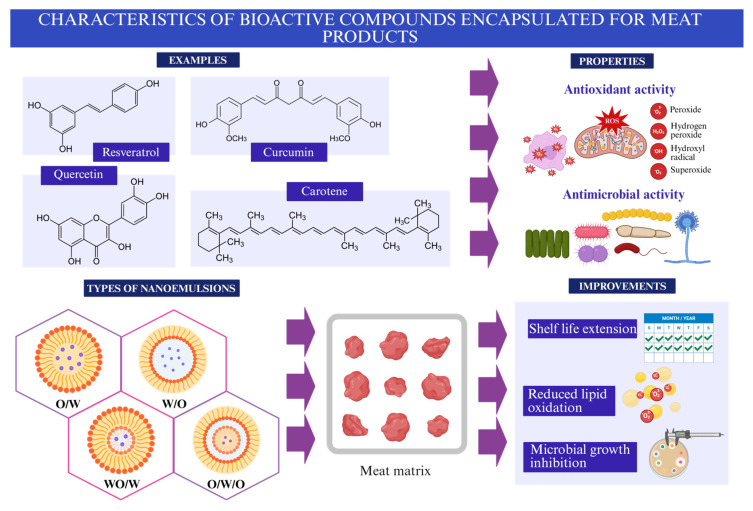
General vision of bioactive compound incorporation into NE for meat product improvement.

Moreover, the direct incorporation of antioxidants and other agents into meat products faces several challenges, such as low stability, reduced bioavailability, and undesirable interactions with components of the food matrix. Consequently, extending their shelf-life while maintaining consumer safety remains a challenge. Therefore, the application of nanoemulsions (NEs) is emerging as a technological alternative to improve meat products [10], as shown in Figure 1. NEs are mostly oil/water (O/W) emulsions that are thermodynamically non-stable but kinetically stable, unlike microemulsions. Their formation demands intensive mechanical energy, ranging from 10^8^ to 10^10^ W/kg [12,13], and there is a thermodynamic force that tends to reduce the contact area between the two phases due to the unfavorable molecular interactions at the oil–water interface caused by the hydrophobic effect [14], which is paramount to reducing interfacial tension to values generally below 10 mN/m to facilitate the formation of stable nanodroplets [15]. Consequently, NEs will always degrade over time by various mechanisms, including gravity separation, flocculation, coalescence, and Ostwald ripening [16]. NEs applied in meat products typically have droplet sizes of up to 500 nm, enhancing stability and bioactive interactions [17]. They have been shown to extend shelf-life by several days, reduce TBARS values, and lower microbial loads by 1–2 log CFU/g [18,19].

This review provides an in-depth analysis of the suitability of incorporating bioactive compounds via NEs into sausages and meat patties formulations to increase the shelf-life of these perishable products. Thus, the objectives of this review include the evaluation of the different techniques for the preparation of NEs, as well as the main factors that limit the incorporation of bioactive compounds in these matrices, and the analysis of the advantages and limitations of this strategy in different studies to determine its industrial feasibility.

## 2. General Aspects of Nanoemulsions

**Figure 2:**
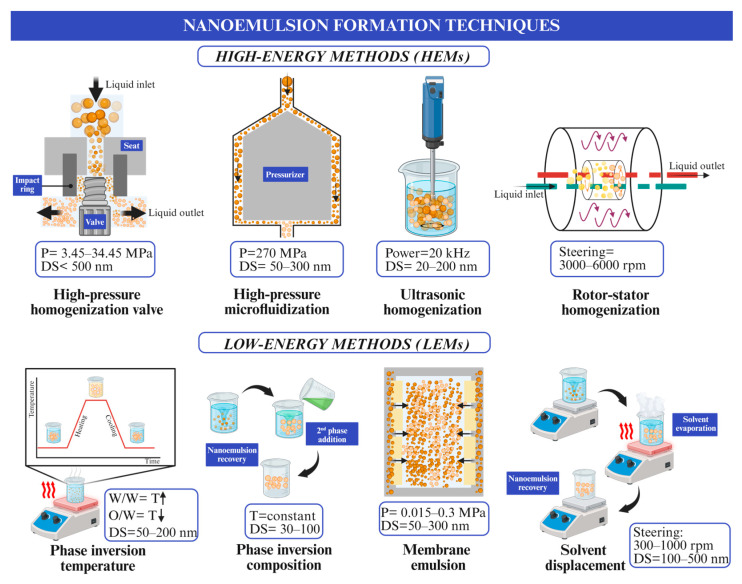
Representation of NE formation by different high-energy and low-energy methods. Upward arrows (↑) indicate increasing temperature, whereas downward arrows (↓) indicate decreasing temperature.

Due to their very small droplets, with an average size of <100–200 nm (although larger systems up to 500 nm with similar behavior have also been reported), NEs have excellent kinetic stability, resistance to particle aggregation, greater physicochemical stability, better dispersion of active compounds, increased bioavailability, and gravity separation [17], as well as increased solubility in the aqueous phase, protection from oxidation of active ingredients and reduction in changes in sensory properties [20]. Thus, NE effectiveness is primarily determined by their droplet characteristics, their physicochemical stability, the composition of the emulsifier, and the method of preparation [21]. Droplet size (DS) distribution determines the extent of kinetic stability, with smaller and more uniform droplets exhibiting lower tendencies for creaming and sedimentation [22]. Additionally, the physicochemical properties of the dispersed and continuous phases, including polarity, viscosity, and ionic strength, modulate interfacial tension and droplet interactions, influencing both storage stability and processing behavior [23]. Consideration of these mechanistic aspects provides a framework for understanding how NEs behave in complex food matrices, independent of the specific bioactive compounds or applications addressed in subsequent sections. This understanding is essential for selecting the most appropriate formation technique, which is commonly classified into high-energy (HEM) and low-energy methods (LEM) according to the amount of energy required for droplet generation [21,24]. The former include high-pressure homogenization, microfluidization, and ultrasonication, while the latter include phase inversion temperature (PIT), spontaneous emulsification (SE), emulsion inversion point, and membrane emulsification [16,24] (Figure 2). Moreover, emerging synthesis techniques include bubble generation at the liquid–air interface and evaporative ripening [13].

Among all these NE techniques, HEMs are more extensively researched and developed on an industrial scale to produce food-grade NEs. This is primary due to the process being faster, since HEMs use mechanical forces, such as shear stress, turbulence, cavitation, and high-pressure application, to break the droplets into nanoscale sizes. This generally produces a smaller DS in a rapid emulsification process. High-pressure valve homogenization (HPH), high-pressure microfluidics (MFH), ultrasonic homogenization (USH), and rotor–stator homogenization (RSH) are commonly considered as HEMs. However, these methods require specialized, and often expensive, equipment, such as high-pressure homogenizers, ultrasonic processors, and microfluidizers [25,26]. Even though HEMs require more energy (~10^8^–10^11^ W/kg) and are relatively costly, they are effective in reaching small DSs [27]. Likewise, HEMs can produce more stable NEs with less surfactant than LEMs. Among these HEMs, HPH and MFH were first used in the dairy industry, being able to reduce fat DS and avoid phase separation. Moreover, MFH has been recently incorporated in the fruit and vegetable industries to produce highly stable juices, since cloudiness, turbidity, and pulp sedimentation are reduced [28].

LEMs are based on physicochemical principles, such as phase inversion, spontaneous emulsification, or interfacial instability, to form NEs [21]. These techniques consume less energy (~103–105 W/kg), are easy to implement, and do not require expensive equipment to manufacture food-grade NEs [29,30]. As they do not require intense mechanical forces, relatively large DSs (30–500 nm) are obtained, making them ideal for heat-sensitive compounds due to the gentler processing conditions [21,31]. However, due to the need to use synthetic surfactants, LEMs have limitations in their food-related applications due to the selection of the appropriate surfactant and oil type [24,32]. Furthermore, despite being generally more scalable and cost-effective, they may require longer processing times [21]. PIT, phase inversion composition (PIC), SE, ME, and solvent displacement/evaporation (SD/E) are commonly used in low-energy techniques [21,31]. Although LEMs have been demonstrated as technically feasible, their application has been studied at lab and pilot scales, with a lack of information regarding industrial application. A more detailed comparison of the different HEMs and LEMs can be found in Table 1.

Beyond their basic preparation and compositional characteristics, NE behavior is strongly influenced by interfacial phenomena and matrix interactions. The interfacial layer formed by emulsifiers not only stabilizes droplets against coalescence and flocculation but also mediates interactions with other food components, such as proteins, salts, and polysaccharides, which can affect rheology, dispersion, and mechanical stability [23]. These interactions can modify interfacial tension, droplet charge, and rheological properties, affecting droplet dispersion, viscosity, and mechanical stability [34].

Collectively, these factors provide the basis for the functional potential of NEs in meat products as a promising strategy to enhance the nutritional quality, acceptance, and shelf-life of these products. However, public acceptance of NEs in the meat industry is currently dependent not only on the benefits described but also on regulatory aspects and consumer perception. Among the concerns are possible adverse effects on health and the environment, making it essential to carry out in-depth studies on the toxicity and biodegradability of these nanostructures [35]. Research must continue to evaluate the safety and effectiveness of these applications, and effective communication strategies must be developed to educate consumers about the benefits of nanotechnology. Therefore, the aim of the present study is to analyze the application of the NEs of bioactive compounds in sausages and meat patties.

## 3. Nanoemulsion Effects on Bioactive Compound Stability and Encapsulation

NEs can significantly enhance the stability and functionality of bioactive compounds by encapsulating them in tiny droplets. This encapsulation creates a protective barrier around sensitive nutrients, helping to preserve their chemical integrity and slow down degradation [36]. In the context of this review, bioactive compounds are mainly referred to as naturally derived compounds that have shown preservative capacities (such as antioxidant and antimicrobial activities) to extend the shelf-life of sausages and meat patties. Thus, bioactive compounds discussed in the following sections include phenolic compounds (e.g., carvacrol, resveratrol, and quercetin), essential oils, and lipophilic antioxidants (e.g., vitamin E), which are typically found in plant extracts.

### 3.1. Factors Affecting Bioactive Compound Stability in NEs

Encapsulating bioactive molecules within NE droplets can protect them against several destabilizing factors, as many bioactive compounds are susceptible to chemical degradation (e.g., hydrolysis or isomerization) during processing and storage. Its mechanism of action consists of isolating the compounds of interest within a dispersed oil or water phase, thus reducing direct exposure to reactive elements, such as water or oxygen [37]. Based on the nature of dispersed and continuous phases, NE can be classified into O/W, water-in-oil (W/O), and multiple emulsions (W/O/W or O/W/O), generally with a preferred DS higher than 200 nm [38]. This value improves kinetic stability by preventing phase separation due to gravitational forces, which contributes to the extended shelf-life of NE-based formulations. Therefore, it is essential to achieve an optimal DS value. This can be accomplished by manipulating phases using different surfactant agents, applying various processing techniques, or altering the proportions of the ingredients [39].

Several studies have demonstrated effective encapsulation strategies, providing concrete examples of how these factors can be leveraged to enhance the stability of NEs in meat derivatives. Most of these formulations have been developed with the primary aim of protecting bioactive compounds against various stressors that commonly induce oxidative degradation, which is a frequent process for fats and oils included in meat formulations (Table 2). For instance, a *β*-carotene (pro-vitamin A) NE coated with a chitosan biopolymer exhibited a dramatically improved resistance to heat and oxygen compared to free *β*-carotene. Free *β*-carotene degraded rapidly when exposed to elevated temperature and UV light, whereas the nanoemulsified *β*-carotene retained about 82% of its content after 3 weeks at 37 °C and nearly 78% after 3 weeks under UV exposure [40]. NE encapsulation mitigates these oxidative effects through three primary mechanisms: absorbing, reflecting, or scattering incidental light; acting as a buffer to stabilize local pH; and providing thermal protection by modulating heat transfer.

Regarding light exposure, especially UV light, NE encapsulation helps mitigate light-induced deterioration by absorbing, reflecting, or scattering incidental light before it reaches the bioactive compound. The extent of photoprotection depends on droplet composition (e.g., opacity of the oil phase or added pigments) and the depth at which the compound is sequestered. Many lipophilic pigments and antioxidants benefit from NE-based light protection [37]. For example, an NE of lycopene extracted from tomatoes was able to protect this highly light-sensitive compound, allowing it to remain bioaccessible after exposure to light and gastrointestinal conditions [36]. In practical terms, this means encapsulated colorants or vitamins in beverages and emulsified foods are less likely to discolor or lose potency under illumination. Another example includes the encapsulation of polyphenols susceptible to isomerization processes that transform them into inactive forms (e.g., resveratrol) in an O/W NE [37]. Similarly, NEs have been reported to improve the chemical stability of other labile phytochemicals, although effective chemical protection often requires a stable interfacial layer, which can also have bioactive compounds [41].

Research has also highlighted the potential of NEs to protect bioactive compounds under acidic or basic environments and during heat treatment. This protective effect is largely due to their capacity to act as a buffer, stabilizing the microenvironment around the bioactive molecules and mitigating the impact of extreme pH or thermal conditions [42]. For example, O/W NE can enclose hydrophobic compounds in oil droplets and therefore protect pH-labile compounds from degradation in unfavorable pH conditions. This form of NE has demonstrated promising results when applied to curcumin, a polyphenol present in turmeric, characterized by its rapid degradation in alkaline conditions, increasing its bioavailability by preventing its premature degradation in the stomach due to acidic pH [36]. NEs can also be formulated to remain stable across a range of pH values common in foods. This pH resilience ensures that encapsulated compounds do not precipitate or degrade when added to acidic media, such as carbonated soft drinks (pH~3) or yogurts (pH~4.5). Similarly, NEs can improve the thermal stability of bioactives by insulating them from direct heat. During processes such as pasteurization or cooking, the small size of NE droplets allows for rapid heat equilibration, avoiding localized hot spots that could char or inactivate the compound [41]. As a result, vitamin-enriched emulsions have shown good retention of vitamins after typical thermal treatments used in beverage processing. For example, vitamin E in an orange oil NE exhibited about 85% retention after pasteurization, indicating minimal loss during heat treatment. These outcomes suggest that NEs can buffer temperature shocks and maintain the integrity of encapsulated compounds during mild heat processing and storage [42].

Moreover, in real food matrices, the stability of the encapsulated compound is largely determined by interactions with proteins, polysaccharides, lipids, and minerals, with the NE structure potentially influencing these effects [43,44]. Thus, certain biopolymers in foods can absorb the droplet surface and reinforce the protective layer. For instance, a protein-rich matrix might contribute to a thick protein coating around droplets, which further inhibits oxidation and compound leakage [45]. However, the food matrix can also pose challenges. High salt or mineral content in a food matrix can screen the electrostatic charges that stabilize NEs, potentially causing droplets to aggregate or coalesce. If an NE flocculates or breaks into the matrix, the bioactive compound may be released prematurely and exposed to degradative reactions. Therefore, the selection of food-grade emulsifiers compatible with the matrix and stabilizers, such as thickening agents, is essential to minimize droplet movement [42].

### 3.2. Factors Influencing the Functionality of Bioactive Compounds in NEs

NEs are widely used in the food industry not only to enhance the stability of added bioactive compounds but also to improve their functionality and bioavailability. This is because NEs can modulate the solubility, bioavailability, and biological activity of the encapsulated compounds, ultimately influencing their effectiveness in food applications [45]. These effects are mediated through several mechanisms, including enhanced solubilization of bioactive compounds, improved absorption in the gastrointestinal tract, controlled and targeted release of encapsulated compounds, and modulation of interactions with biological membranes (Table 2).

**Table 2:** Summary of nanoemulsion encapsulation advantages for various bioactive food compounds.

NE Type	Bioactive Compound	Bioactivities	Factors Affecting Application	Key Findings	Ref.
**Solubilization**					
W/O	Vitamin E	AO, supports immune and skin health	Susceptible to oxidation, heat, or light; hydrophobic, so difficult to disperse in low-fat foods	Prevention of degradation; facilitates incorporation into foods and beverages	[42]
W/O	Essential oils	AO, AM	Susceptible to oxidation and off-flavor	↑ shelf-life and bioavailability	[46]
C	Polyphenols	AO, AM, AI	Appearance; film acceptability	↑ shelf-life and bioavailability	[47]
O/W	Essential oils	AO	Dispersion of oil	↑ tenderness	[48]
W/C	*β*-Carotene	AO, COL	Susceptible to oxidation and heat-induced isomerization	↑ antioxidant and sensorial acceptance	[40]
O/W	Carvacrol	AO, AM	Stability	↑ shelf-life and bioactivity	[18]
**Improved absorption**					
AG-NE	Andrographolide	AI	↓ water solubility	↑ bioavailability	[49]
Starch	Chrysin	AI	↓ water solubility	↑ bioavailability and permeability	[50]
Starch	Quercetin	AO, AI	Susceptible to oxidation; ↓ solubility	↑ antioxidant and bioavailability; stable at↓ temperatures	[41]
Starch	Coenzyme Q10	AO	↓ stability	↑ bioavailability	[51]
O/W	Lipophilic substances	AM	↓ solubility	↑ bioavailability	[52]
**Controlled/sustained release**					
O/W	Curcumin	AO, AI	Sensitive to pH, light, and heat; ↓ solubility	pH and light protection; resistance to gastric conditions; ↑ bioavailability	[36]
O/W	Lycopene	AO, PA	Sensitive to light and heat	↑ antioxidant and bioaccessibility	[36]
W/O	EO	AM, AO	Susceptible to oxidation and off-flavor	↑ antioxidant and shelf-life	[53]
O/W	DHA/EPA FAs	CP, AM	Susceptible to oxidation and off-flavor	↑ antioxidant and sensorial acceptance	[45]
O/W	Resveratrol	AO, CP	Isomerize to inactive form; ↓ solubility and metabolism efficacy	↑ antioxidant and bioavailability	[37]
O/W	Essential oils	AO, AM	Susceptible to oxidation and off-flavor	↓ dose; ↑ release control	[54]
G/C	Essential oils	AO, AM	Susceptible to oxidation and off-flavor	↑ shelf-life and sensory acceptance	[55]
**Interaction with biological membranes**					
O/W	Lipophilic substances	AM	↓ solubility	↑ bioavailability	[52]
O/W	Lipophilic substances	AM, AO	↓ solubility and stability	↑ bioavailability	[52]
O/W	*β*-carotene	AO	Toxicological risks	↑ bioavailability and solubilization	[56]

Abbreviations: NE—nanoemulsion; C—chitosan; G/C—gelatin/chitosan; AO—antioxidant; PA—pro-vitamin A activity; CP—cardioprotective; AI—anti-inflammatory; AM—antimicrobial; DHA—docosahexaenoic acid; EPA—eicosapentaenoic acid; FAs—fatty acids; ↑—high; ↓—low.

Regarding solubilization, many bioactive nutrients and phytochemicals have limited solubility in either water or oil, which constrains their use in certain foods. NEs offer a strategy to increase the apparent solubility of poorly soluble compounds by dispersing them as tiny droplets. For example, O/W NEs of hydrophobic compounds (e.g., lipophilic vitamins) prevent these compounds from aggregating or separating in aqueous systems, enabling their delivery as stable microscopic oil droplets throughout the food matrix [42]. This approach also preserves the color of the product [42], as the small size of the NE droplets allows for the incorporation into meat products without noticeably altering their appearance or texture. These effects would only be observed with DSs below 100–200 nm, which significantly improve the interaction of lipophilic bioactives with the aqueous phase of meat matrices, overcoming the limitations of pure oils (low solubility, volatility, and poor stability) [46]. Other studies reported that chitosan-based NEs in muscle foods are a viable approach to deliver natural plant-derived bioactives in processed-meat products, improving their solubility and functional performance while ensuring physicochemical stability during storage [47]. These findings indicate that NE systems can serve as effective vehicles to improve the functional performance of bioactive compounds in meat products.

Beyond solubilization, NEs can improve the absorption of bioactive compounds in the gastrointestinal tract. For example, in a study using an NE formulation loaded with andrographolide (AG), researchers observed a dramatic increase in intestinal uptake; the optimized AG-NE (DS~122 nm) displayed ~8.2-fold greater absorption in the jejunum compared with a standard suspension, resulting in a ~594% increase in overall oral bioavailability [49]. Similarly, formulations using NEs have been shown to improve the bioaccessibility and permeability of poorly soluble polyphenols, such as chrysin [50]. However, there are currently no available studies evaluating the improvement in absorption of these formulations after their incorporation into meat products. Nevertheless, previous studies conducted with other compounds demonstrate that NE-based systems can significantly improve the bioaccessibility and bioavailability of lipophilic compounds after gastrointestinal digestion [51]. Therefore, given that NEs successfully enhance the dispersion and stability of lipophilic actives in meat matrices and that their small DS favors the efficient digestion and micelle formation under simulated gastrointestinal conditions, it is plausible that similar improvements in absorption could be obtained when these NEs are incorporated in meat-derived foods.

Moreover, recent advances suggest that NEs and related lipid- or polymer-based nanocarriers may enable the controlled and sustained release of encapsulated bioactive compounds when applied to meat and meat products. For example, NEs of essential oils in lipid or biopolymer meat products can form modified release systems that gradually deliver the active compounds over time, reducing rapid volatilization or burst release that typically limits the efficacy of free oils [54]. In fact, case studies carried out with coatings or films loaded with an NE of essential oils applied on meat surfaces (e.g., in sausages, patties, or ready-to-eat products) have demonstrated prolonged antimicrobial or antioxidative action during storage, consistent with a slow-release profile of the bioactive compounds [55]. This gradual release can also be observed in the digestive tract, where it has been observed that NEs formulated to resist stomach conditions, remaining intact in acidic pH and in the presence of gastric enzymes, are able to release bioactive compounds in the small intestine [36]. This ensures the compound is taken up in the optimal part of the gastrointestinal tract and possibly via specific uptake pathways [36]. Thus, NE-based systems emerge not only as stabilizing carriers but also as delivery platforms enabling controlled, time-extended release, a property particularly useful in meat preservation, where extended shelf-life and maintenance of bioactive efficacy are desired.

In addition to time-controlled release, recent evidence indicates that NEs may enhance the interaction of encapsulated lipophilic bioactive compounds with biological membranes, thereby facilitating their uptake and potentially improving their biological efficacy. This capacity is attributed to the small diameters of NE droplets (typically below 200 nm), which gives rise to a high surface area-to-volume ratio, which maximizes interfacial contact between the lipid droplets and cell membranes, a feature that has been hypothesized to promote passive diffusion or the endocytosis of NEs [52]. This theory is supported by different studies. For example, NEs with DSs in the range of 45–120 nm were shown to be taken up by intestinal epithelial cells, with a portion of the NE material traversing the cell monolayer, indicating that at least part of the material may cross the epithelial barrier via transcellular transport [56]. Nevertheless, the successful translation of this mechanism from model systems (liquid foods or in vitro cell models) to complex matrices, like processed meat, requires further investigation, as the food matrix, digestion process, and physicochemical properties of the NEs may modulate membrane interactions, droplet stability, and ultimately uptake and bioavailability.

## 4. Application of Nanoemulsions in Sausages and Meat Patties

### 4.1. Impact of Nanoemulsions on the Physicochemical Quality of Meat Products

The incorporation of NEs into meat products has been shown to exert beneficial effects on key physicochemical parameters during storage (Table 3). For instance, oil-in-water NEs added to pork patties significantly reduced cooking and thawing losses and improved water-holding capacity, while also enhancing texture and color stability compared to conventional oil- or water-based formulations [45]. In this case, the improvement of the techno-functional properties of the patties was produced due to the matrix structure obtained by the incorporated O/W NEs instead of O/W emulsions. These results are in line with those obtained in a more recent study, in which hydrocolloid NE coatings applied to poultry filets increased their water-holding capacity and showed smaller increases in shear force over storage than untreated controls, indicating a better preservation of texture and reduced moisture migration. However, these improvements are linked to the nanoencapsulated aloe vera gel and hemp seed oil, not only the NE incorporation into the patties [57]. In another study, an NE-based edible coating (rosemary extract and ε-poly-l-lysine NE) applied to fresh meat enhanced color stability and reduced drip loss during refrigerated storage, effects the authors attributed to the coating’s fine droplet structure and improved surface film formation [58]. NEs with crodamol can act as an ultrahydrophobic active ingredient, avoiding Ostwald ripening and achieving a high rate of long-term stability [59]. These reductions in water losses and water activity attributed to NEs reduce the chances of microbial growth [60]. However, to observe this property, it is necessary to store meat products at an optimum temperature of 4 °C. Higher temperatures increase the Ostwald ripening rate as NE droplets move more frequently in the continuous phase, and their chances of collision increase. In addition, higher temperatures result in larger DS values, as the diffusion of small oil droplets through the continuous phase causes them to re-deposit on larger oil droplets to form larger particles [61,62]. Further advantages of NEs include improved physicochemical properties and masking the taste or odor of the core material [63]. Therefore, the incorporation of NEs in meat products can improve water-holding capacity, reduce cooking and thawing losses, and support greater stability of pH and color compared to untreated controls. These effects are generally attributed to the fine DS and uniform dispersion of the NE, which enhance interactions with meat proteins and limit moisture migration [23].

### 4.2. Use of Nanoemulsions as Natural Preservatives in Sausages and Meat Patties

Meat and meat products are considered one of the foods most susceptible to spoilage, generating large losses in the meat industry [92], with over 40% of these losses occurring at the retail and consumer stages in developed nations [93]. The decline in meat product quality is a consequence of their chemical composition, characterized by a high moisture content and a large percentage of unsaturated FAs, which makes meat and its derivatives a perishable food due to high microbial growth, high susceptibility to lipid peroxidation, and enzymatic autolysis phenomena [94,95]. This spoilage is even greater in meat products such as sausages or patties [96]. This is primarily due to the handling of meats from diverse sources, resulting in a loss of structural integrity and increased surface area. This handling increases the risk of microbial contamination and increases exposure to oxygen, which promotes oxidative processes. This is especially relevant in products with a high fat content, as they are particularly susceptible to lipid oxidation [96]. Moreover, this lipid oxidation generates various genotoxic and cytotoxic compounds that represent a risk to the health of consumers [94].

All these factors make it necessary to add preservatives to the formulation of these meat products, which means that practically all of them contain a significant amount of preservatives in their formulation [96]. Among the most frequently used additives are those derived from chemical synthesis. Key representatives of this group are butyl-hydroxy-anisole (BHA), sulfur dioxide, butyl-hydroxy-toluene (BHT), tert-butyl-hydroquinone (TBHQ), propyl-gallate (PG), nitrites, nitrates, ascorbates, monosodium glutamate, and liquid smoke. These compounds have proven to be highly effective, but their consumption is often associated with adverse health effects [92]. Among the reported adverse effects are asthma, urticaria, abdominal pains, nausea, diarrhea, seizures, anaphylactic shock [97], hyperactivity, liver damage, carcinogenesis, and mutagenesis [92]. Particularly relevant is the use of BHT and BHA in meat formulations, which are associated with an increased risk of cancer, leading to their restriction [97].

Consequently, recent scientific research has primarily focused on finding natural compounds with properties that can replace these chemical additives. These substances are referred to as bioactive compounds and can be extracted from microbial, plant, and animal sources, including their waste [98]. Among these, plant by-products and algae are the most economically viable sources [99], offering the added benefit of reducing costs and environmental issues by utilizing underexploited raw materials [95]. This has led to a projected market value of USD 47.50 billion by 2023 [100]. Some practical application examples include bioactive compounds extracted from plants, such as grapes or rosemary, as well as those derived from animals, like chitosan from fish, which have demonstrated strong antioxidant and antimicrobial properties [98]. The incorporation of these bioactive compounds also improves nutritional composition, taste, and oxidative stability, and protects consumers from free radicals that can cause chronic diseases [92]. In fact, the addition of bioactive compounds in meat products (Table 4) has proven to be one of the most effective ways to extend shelf-life by maintaining physicochemical quality, improving microbial safety, and increasing oxidative stability in processed-meat products [10,58]. Phenolic compounds are among the most studied natural preservatives, including phenolic acids (i.e., caffeic acid, gallic acid, and rosmarinic acids), flavonoids (i.e., catechin, kaempferol, and quercetin), and phenolic dipterans (i.e., carnosol and carnosic acid). In addition, considerable attention has been given to the use of volatile oils (i.e., menthol, eugenol, thymol, carnosol, and carvacrol) [99,101] and pigments (i.e., curcuminoids, carotenoids, and betacyanins) [99]. In the future, it will be necessary to examine the phytochemical profile of plants that have been used in traditional medicine and cooking for generations to determine their feasibility in meat product preservation.

#### 4.2.1. Effect of Nanoemulsions on Oxidative Stability and Shelf-Life

Meat products are mainly composed of proteins and lipids. The lipidic composition of these products is susceptible to degradation, with lipid oxidation being the main nonmicrobial cause of quality deterioration. This deterioration process not only affects the nutritional values of meat products, reducing the content of essential FAs and lipidic vitamins, but also affects the sensorial characteristics, causing rejection by the consumer [8]. Moreover, both sausages and meat patties are subjected to various treatments that increase the risk of spoilage. These treatments include the addition of pro-oxidants, such as salt, to meat product formulations [140]. This, combined with exposure to air and the reduced efficacy of endogenous enzymes (glutathione peroxidase, catalase, and superoxide dismutase) and non-enzymatic components (i.e., ascorbic acid, spermidine, spermine, glutathione, α-tocopherol, carnosine, and lipoic acid), makes these products more susceptible to deterioration and loss of quality [140]. This loss of quality leads to a decrease in sensory acceptance due to the appearance of unpleasant odors, loss of color, and the production of gas and slime. Furthermore, alterations in nutrient metabolism occur, notably resulting in the formation of harmful toxic aldehydes [94,99,140], making it necessary to improve storage conditions, which often involves the use of refrigeration temperatures [96].

To combat this problem, the meat industry incorporates antioxidants to retard or prevent oxidative reactions [141]. In this way, EOs have been studied as potential antioxidant ingredients, with NEs being presented as excellent formulations to incorporate these compounds since they can improve solubility, bioavailability, and antioxidant activity [10]. For instance, the incorporation of a curcumin NE into Harbin red sausages at different concentrations (0.05, 0.1, and 0.15% of curcumin) showed significantly lower TBARS values than the control after 6 days of storage, with 0.8 mg/kg for the control and 0.5 mg/kg for the 0.15% curcumin NE (Table 4). Authors attributed the potent antioxidant activity of the NE to the effect of curcumin as a potent single-linear oxygen quencher, being able to regenerate secondary antioxidants, therefore, intercepting lipid free radicals from forming phenoxy within the cell membrane [67]. In another study, the incorporation of a *β*-carotene NE into goat meat sausages at different concentrations (10, 20, and 30 g/100 g) led to better oxidative stability compared to the control, although no significant differences in TBARS were observed between the *β*-carotene NE-added sausages, demonstrating comparable effects on secondary oxidative inhibition of the *β*-carotene-NE at the concentrations studied [68]. Similar results were obtained with a Tunisian thyme essential oil NE (3.5%) incorporated in fresh bovine meat, which showed lower values of TBARS and TVB-N than the control (0.3% and 5 mg/100 g less, respectively) [69]. Regarding meat patties, the incorporation of cinnamon EO and rosemary extract NEs into chicken breast patties decreased TVB-N and TBARS values compared to control samples by 7 mg/100 g and 8 mg/100 g, respectively [77]. Due to their effectiveness in reducing hydrogen peroxide-induced discoloration, NEs are promising candidates for ensuring the color stability of meat products [18]. Therefore, the incorporation of natural compound NEs into meat products presents a viable manner to preserve the products’ quality by stabilizing the matrix and, therefore, extending their shelf-life.

#### 4.2.2. Effect of Nanoemulsions on Microbial Safety and Preservation

Meat provides an ideal environment for microbial growth due to its rich nutrient content, high water activity (a_w_), and low acidity [46]. Microbial contamination of meat and meat products accelerates spoilage, shortens shelf-life, and compromises quality, posing a significant public health risk and leading to substantial economic losses. To address this issue, various food additives have been incorporated into meat products. In recent decades, there has been growing interest in natural antimicrobial compounds, with nanoencapsulation emerging as an effective strategy to enhance their stability, bioavailability, and uniform dispersibility within the product [76]. In this way, several studies have focused on the incorporation of different natural antimicrobial formulas, such as EOs and extracts, into meat products like sausages and patties. For instance, an O/W NE using capsaicin as an antimicrobial ingredient was incorporated into Merguez sausages. The incorporation of this NE led to a high antimicrobial activity against Gram-positive bacteria, such as *Listeria monocytogenes*, and *Staphylococcus aureus* (surface IZ = 200–400 mm^2^), and moderate antimicrobial activity against Gram-negative bacteria, such as *Escherichia coli* and *Salmonella arizone* (surface IZ≤ 200 mm^2^). Thus, sausages with NEs incorporated showed a shelf-life of 20 days when refrigerated at 4 °C (Table 4) [70]. Similar results were obtained after incorporating thyme EO NEs into meat products, where they extended shelf-life from 7 to 20 days at 4 °C, effectively reducing the growth of lactic acid bacteria, yeast, and mold strains [69]. In a different study, the potential antimicrobial activity of thymol NEs was assessed in a sausage model. Researchers inoculated three bacteria, *S. aureus*, *E. coli*, and *C. perifringens*, and incorporated thymol and thymol NEs to investigate their effect on the sausage model. Results showed that MIC and minimum bactericidal concentration (MBC) values of sausages with thymol (600 mg/kg) were almost twice as high as the MIC and MBC values of sausages with thymol NEs for the three bacteria inoculated, which showed the higher antimicrobial effect of NEs, extending the sausages’ shelf-life for 30 days at 4 °C [75]. This reduction in microbial growth has also been seen in minced meat/meat patty products. For instance, chitosan NEs using *Mentha piperita*, *Punica granatum*, *Thymus vulgaris*, and *Citrus lemon* EOs at different concentrations were incorporated into minced beef. Authors determined the antimicrobial potential of these NEs with in vitro tests against *E. coli* and. *S. typhimunium* and in vivo tests storing the product for 10 days at 4 °C, showing excellent results extending the meat’s shelf-life [72]. Thus, oil-based NEs are proven to exert antimicrobial action against Gram-negative and Gram-positive bacteria [142]. The biological activity of these EOs can be improved by increasing the surface area, which allows for the use of lower doses of EOs [63]. Moreover, the use of edible coatings that not only protect meat products from mechanical damage and selective chemical reactions but also control the transport of antibacterial agents and preserve their bioactivity is a relevant issue for optimizing the quality of an NE after its development with natural active ingredients [143]. However, despite the positive results obtained by incorporating different compounds and EOs as NEs in sausages and patties to inhibit microbial growth, it is important to consider the positive and negative impacts on sensorial characteristics.

### 4.3. Impact of Nanoemulsions on Functional Properties of Meat Products

Although antioxidant and antimicrobial effects are the primary roles of bioactive compounds in meat products, they simultaneously provide other significant nutritional and health benefits beyond basic nourishment. This places bioactive compounds at the forefront of consideration when formulating functional meat products with comprehensive nutritional value [144]. Healthier meat products with NE-encapsulated bioactive compounds can be developed through NEs without detrimental effects on the sensory characteristics or shelf stability of the products [80,86]. The type of NE influences the encapsulation efficiency of bioactive compounds and their release profile in food matrices. O/W NEs are the most widely used in food-related applications due to their ability to encapsulate hydrophobic compounds in an aqueous environment [145]. Active phytochemicals and antioxidants can be added to meat products by NEs that help incorporate components with low polarity. These include essential oils, phytochemicals like curcumin, resveratrol, lycopene, and even hydrophobic nutraceuticals, such as fat-soluble vitamins (A, D, E, and K), carotenoids (*β*-carotene, lycopene, lutein, and zeaxanthin), catechins, and flavonoids. Moreover, the nanoemulsified components can enhance the meat products’ nutritional value, as well as improve texture, flavor, sensory appeal, and shelf-life [146,147].

Processed meat products like sausages, patties, nuggets, and surimi can be conveniently enriched with omega-3 FAs by blending NEs directly into the ground meat mixture [146]. The incorporation of omega-3 polyunsaturated fatty acids (PUFAs)—particularly alpha-linolenic acid (ALA) from plant-based sources, such as chia oil and linseed oil—into meat products addresses the imbalance between omega-6 and omega-3 FAs in industrialized diets. These FAs play crucial roles in brain development, inflammation reduction, and cardiovascular health [148]. For instance, as a rich source of omega-3 PUFAs (EPA and DHA), fish oil was effectively added to Spanish bologna-style sausage, resulting in an improved omega-6 to omega-3 FA ratio [149].

Fat-soluble vitamins, particularly tocopherol and vitamin D, serve as a valuable functional nutrient because of their strong antioxidant properties, functioning as a bioactive compound in meat products. The nanoemulsified form of tocopherol has been effectively utilized in fish sausages made from golden pomfret filets, at concentrations of 250 or 500 mg NE/kg. During a 16-day refrigerated storage period (4 °C), tocopherol NEs helped preserve PUFAs, inhibited lipid oxidation, and improved the quality of the product while keeping desirable texture attributes. This antioxidant activity of nanoencapsulated tocopherol is produced by interrupting the free-radical oxidation chain reaction from forming tocopheroxyl radicals and, therefore, interrupting lipid peroxidation propagation [147]. Vitamin D3 (cholecalciferol) plays a significant role in bone health, immune function, and cellular processes, yet there is a global concern about its declining levels. Tocopherol NEs were helpful tools for tracking PUFAs under refrigerated storage (4 °C) over a 16-day period; the NEs inhibited lipid oxidation and improved product quality while keeping desirable texture attributes [8]. Considering the socio-economic impact of vitamin D3 deficiency, capturing the opportunity to forge appealing and functional fortified foods by leveraging the food market with livestock products fortified using vitamin D oil-in-water NEs has proven effective [67]. With meat and meat products being part of a daily diet, enriching them with vitamin D3 presents a product that meets public demand while addressing several health issues. Developments in nanotechnology, in particular, nanostructured oil-in-water vitamin D NEs derived from natural ingredients, like safflower oil and pea protein, promote nutrient retention and bioavailability while guaranteeing stability during processing, which was the primary goal. Notably, optimized NEs have demonstrated 78% vitamin D3 retention in cooked beef patties, compared to just 31% for non-encapsulated forms. These systems not only ensure nutrient retention during processing and storage, but also help produce functional meat products, which parallel consumers’ interests in eating health-promoting, less processed foods [89]. Selenium (Se) is an essential trace element required for health and growth and may be delivered by Se-enriched NEs. This supplement, involved in antioxidant defense systems and thyroid hormone metabolism, represents another way to enhance the functional nutrient profile of meat products through NE technology. These NEs have been shown to prolong the shelf-life of chicken sausages from 20 to 30 days, displaying their promising role in increasing product quality [76].

Polyphenolic compounds, including flavonoids, phenolic acids, and stilbenes, are a major class of bioactive compounds incorporated into meat products via NEs. The functional enrichment of Harbin red sausage via curcumin with NE (Cur@QCS/SA) using chitosan quaternization and sodium alginate stabilization was investigated. Curcumin is a plant-based polyphenol that exhibits antioxidant, anti-inflammatory, and antimicrobial characteristics. Additionally, it is significant that curcumin may be administered using the NE system, and the latter has significantly improved the stability and bioavailability of curcumin. The incorporation of 0.15% Cur@QCS/SA showed an improvement in key quality parameters, such as pH, moisture, color, texture, and lipid oxidation profile, while also maintaining favorable sensory characteristics. The observed improvements are the result of the synergistic antioxidant and antimicrobial mechanisms of curcumin and the water immobilization capacity of chitosan and sodium alginate. Specifically, the radical scavenging ability of curcumin leads to a reduction in lipid oxidation reactions, which slows down pH reduction as fewer aldehydes and acids are formed. At the same time, the antimicrobial capacity of curcumin is related to the alteration of the bacterial membrane. Finally, water immobilization produced by chitosan and alginate nanostructures provides a resistant polysaccharide matrix capable of improving water-holding capacity and reducing water loss. These findings provide further evidence for the use of curcumin NEs as an option for developing meat products with functional nutritional properties [67]. The novel approach of using NEs for the delivery of functional nutrients increases the nutritional value of meat, giving health benefits to the consumer with no change to the sensorial characteristics [147]. Taste can be maintained as the nutritional value is improved and shelf-life is extended by utilizing lower bioactive dosage requirements provided by the nanoencapsulation of plant bioactives, including eugenol and phenolic acids. The nutritional value of meat proteins may decline in storage when vital amino acids are oxidatively damaged, as well as through processes that reduce overall meat digestibility. Protein oxidation is mostly evaluated through the accumulation of carbonyl compounds, which is often initiated by direct attack from reactive oxygen species or other more advanced forms of lipid oxidation, like aldehydes and ketones [150]. Recent studies have shown that incorporating nanoemulsified bioactive compounds, such as resveratrol (RES) and clove (CEO), into edible films can turn these processes around. In minced camel meat, the rate of carbonyl content increase over 20 days was significantly lower in untreated samples in comparison to those treated with NE-based basil seed gum films containing CEO and RES, suggesting a protective effect on protein structure. The antioxidant activity of the phenolic compounds in RES and CEO, specifically in maintaining the activity of more reactive species and the preservation of the sulfhydryl group, is exceptionally relevant to maintaining protein functionality. These results demonstrate the effectiveness of the use of nanoencapsulated polyphenols not only to increase oxidative stability but also to preserve proteins and their functional and nutritional properties in meat products [80]. Utilizing bioactive-rich NEs in meat systems corresponds with more recent dietary trends, as they support the development of functional meat products that provide high-quality nutrition and support consumer health [147]. Further research should examine optimizing encapsulation strategies, long-term stability properties, and nutritional and sensory evaluations.

## 5. Limitations of Incorporating Bioactive Compounds: Consumer Acceptance and Perception

**Figure 3:**
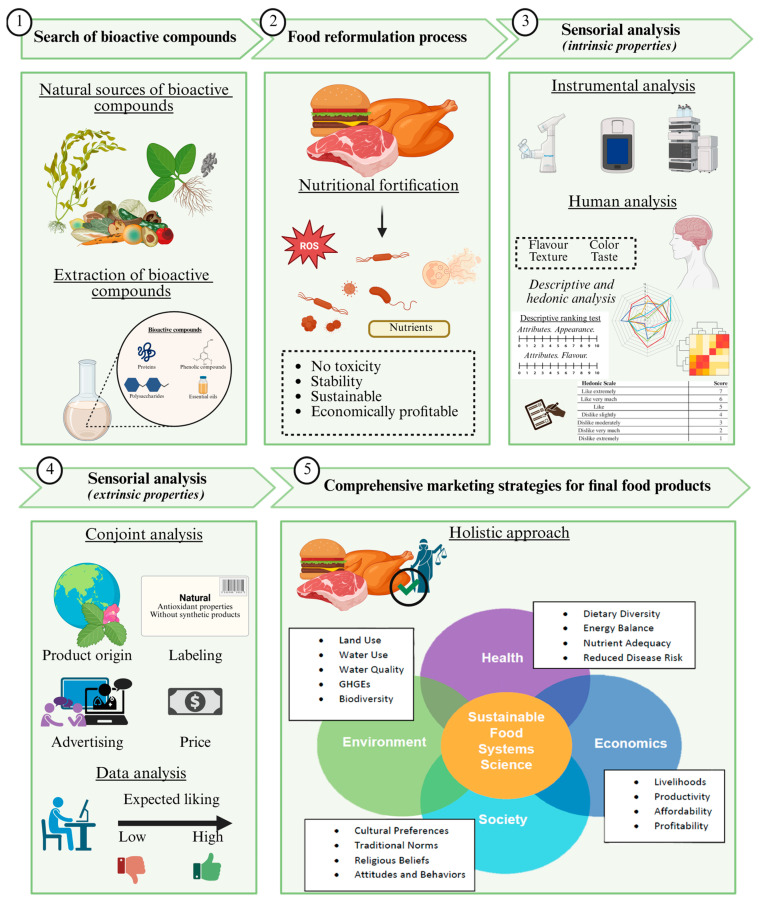
Challenges in product development of bioactive compounds in NEs: consumer acceptance and perception.

The alteration of the organoleptic properties of meat products following the incorporation of bioactive compounds as additives is a topic of great interest in food science. This limitation arises from the fact that the direct incorporation of bioactive compounds into sausage and patty formulations often results in undesirable modifications of their organoleptic characteristics [112,151]. The primary sensory attributes potentially affected include taste, aroma, texture, and color (Figure 3).

Changes in taste and smell are mainly due to the nature of the bioactive compounds, which, although they have proven to be effective in preventing rancidity in meat products, in high concentrations can impart a metallic or astringent taste to the products in which they are incorporated [152,153]. On the other hand, certain bioactive compounds, especially when they include volatile compounds such as EOs, have their own flavor and aroma that can negatively influence the sensory perception of meat [154,155]. For example, extracts made from aromatic plants significantly alter the aromatic profile of the foods in which they are incorporated, giving them a herbal character, which is not always accepted by consumers, especially in high concentrations [112,124,156]. Therefore, it is necessary to investigate the optimal concentrations of added oils and spices to mitigate potential adverse effects on sensory properties. This optimization would allow for the development of healthier products with a longer shelf-life and greater consumer acceptance. This is the case with the addition of oak wood and cherry powder extracts in pork patties, or pomegranate peel and pomegranate juice extracts in cooked chicken patties, which increased their sensory acceptance [120,122,157]. Significant differences in aroma, flavor, or overall acceptability were not detected in low-salt sausages upon the addition of garlic derivatives [158] or citrus juice-processing by-products into Bologna sausages [159]. Furthermore, the incorporation of these citric by-products has the advantage of reducing residual nitrite levels, thus avoiding the possible formation of nitrosamines and nitrosamides [159].

In addition, some of these bioactive compounds are pigments that can alter the color of the product to which they are added [160]. Most of the available studies show a positive impact after the incorporation of these compounds due to the increase in the color stability of meat derivatives; in some cases, they increased the intensity and luminosity of the products, which are crucial aspects for consumer acceptance [108,109]. The use of anthocyanins, reddish pigments present in many agricultural by-products such as grape pomace, is particularly noteworthy. These anthocyanins are used in the food industry due to their antioxidant capacity [161,162]. This antioxidant capacity has led to research into the use of anthocyanins as a replacement for sulfites used in the meat industry [163]. However, its use must be studied for each product since its incorporation often causes a darkening of the product, which could alter the acceptance of the product by consumers [164]. This color alteration would not affect other organoleptic properties such as juiciness, crispness, oiliness, saltiness, and flavor [165]. Another example includes the addition of clove extract to cooked and refrigerated beef patties, which increased the stability of the product’s red color, maintaining sensory characteristics for 10 days [166]. Similar effects were noted upon the addition of rosemary extract to pork burger formulations and oregano extract to lamb burger formulations [167,168]. In another study carried out with green tea extract, it was observed that the addition of this extract improved color preservation and prevented the appearance of rancid flavors in beef patties, without undesirable modifications in the odor, taste, and texture of the patties [169]. Therefore, the direct incorporation of these bioactive compounds does not always lead to a decrease in the acceptability of the product.

Another critical consideration in the reformulation of meat products is the impact of incorporated bioactive compounds on texture. This becomes particularly important when using proteins or polysaccharides, as these compounds are characterized by gelling properties and, therefore, can alter the consistency of products, such as sausages and patties [170,171]. In some cases, these textural changes can be beneficial since they increase water retention, with the consequent increase in the juiciness of the products. However, an excess can cause a decrease in firmness or an undesirable change in the elasticity of the product [172]. For example, it has been observed that the incorporation of vegetable proteins (i.e., pea protein) in meat products can increase tenderness and juiciness, but, in some cases, they can diminish the chewiness or firmness that is typically desired in products such as sausages [173]. These are not the only additives that can have an impact on texture, as numerous studies have shown that the addition of phenolic compounds can increase the stickiness of products, causing rejection by consumers [174,175].

In all cases, the available studies suggest that the impact of bioactive compounds on organoleptic properties is not uniform and depends on several factors, such as additive concentration, additive extraction method, meat product type, processing method, and consumer preferences. For example, it has been observed that pulse electric fields (PEFs) do not generate off-flavors in volatile oils [176]. However, emergent techniques are generally associated with high equipment costs, which can limit their economic feasibility for industrial applications. Beyond the cost of production, scalability represents another major challenge [177,178]. Many laboratory-scale methods (e.g., ultrasonication or microfluidization) are difficult to translate directly to industrial lines due to differences in batch size, energy distribution, and process control. In addition, maintaining uniform quality across large production batches is complex, as slight variations in temperature, pH, or raw material properties can significantly affect emulsion characteristics [45,179,180]. In fact, one of the main problems is the standardization of natural extracts, since their composition in the different plant matrices would depend on edaphoclimatic factors [100]. Storage, transport, and integration into existing meat-processing workflows also require additional infrastructure, such as controlled temperature environments and specialized mixing systems, which further increase the costs [181,182]. Furthermore, the use of emerging techniques, such as encapsulation and active packaging, are promising strategies for incorporating bioactive compounds into meat products like patties and sausages, as they improve sensory acceptability by reducing extreme flavors associated with many bioactive compounds [183]. Encapsulation improves the stability and controlled release of bioactive compounds, safeguarding them from degradation throughout processing and storage [184]. Active packaging further enhances product performance by gradually releasing antimicrobial or antioxidant agents, which help to prolong shelf-life and maintain sensory qualities [164]. However, both techniques introduce additional design, handling, and cost considerations [185]. Finally, the economic benefit of using NEs must be weighed against potential increases in retail price, consumer willingness to pay, and competition with conventional preservatives [100,181,182]. Addressing these operational and economic barriers is critical to ensure that NE technologies can move from promising laboratory studies to practical, cost-effective applications in the meat industry.

Therefore, scientific research has shown that, with the appropriate formulation and a balanced amount of additives, it is possible to improve the functional properties of meat products without unduly compromising their organoleptic characteristics. However, this requires a detailed and specific approach for each type of meat product. Such an optimization must be accompanied by a careful evaluation of safety, toxicological aspects, and regulatory compliance. In the case of bioactive NEs, formulation parameters not only affect organoleptic properties but also influence system stability, the release of encapsulated compounds during gastrointestinal digestion, and their subsequent bioavailability. Although these systems are generally based on food-grade ingredients (e.g., emulsifiers such as lecithin, polysorbates, proteins, or polysaccharides), their nanoscale structure raises additional questions regarding long-term exposure and repeated consumption, which highlights the need for specific toxicological evaluation [45].

Current scientific evidence suggests that NEs tend to be destabilized during gastrointestinal digestion, leading to the release of encapsulated bioactive compounds. While this behavior supports their functionality, information regarding their long-term safety, chronic exposure, and interaction with complex meat matrices remains limited [185,186]. Most available studies focus on short-term efficacy rather than comprehensive in vivo safety assays. Moreover, these assays are commonly conducted under simplified laboratory conditions, which do not fully replicate the stresses encountered during industrial processing. These analyses should consider the different mechanical and thermal treatments (e.g., chopping, mixing, cooking, smoking, freezing, or extended storage) that sausages and patties undergo that can significantly affect NE stability and the retention of bioactive compounds [10,46,187]. For example, high shear forces during mixing or chopping can cause droplet coalescence or partial breakdown of NEs [10]. On the other hand, heating and smoking may accelerate lipid oxidation or degrade sensitive phenolic compounds, reducing antimicrobial and antioxidant efficacy [45,188]. Freezing and thawing cycles can promote phase separation, particularly in NEs stabilized with low-molecular-weight surfactants [189,190]. Current evidence suggests that protein- or polysaccharide-stabilized NEs tend to better resist these processing stresses, maintaining droplet integrity and functionality [191,192,193]. However, systematic studies comparing different NE formulations under realistic industrial conditions are scarce. The lack of standardized protocols for assessing NE behavior during complex meat-processing operations represents a major gap, limiting the ability to predict performance and optimize formulations for large-scale application. Addressing these gaps is essential to ensure that the functional benefits of NEs observed in laboratory studies are maintained in commercial meat products.

From a regulatory perspective, although individual components of NEs are often approved for food applications, nano-structured delivery systems are not always explicitly addressed within existing regulatory frameworks, and specific guidance on the use of NEs in processed-meat products is still evolving [10,194]. Moreover, regulatory requirements and definitions vary between regions. For example, in the European Union, nanomaterials used in foods are subject to specific evaluation requirements, particularly when their nanoscale properties may alter absorption, distribution, or biological interactions, emphasizing the need for case-by-case risk assessment, considering particle size, surface properties, stability, and behavior during digestion [195,196]. Another important regulatory aspect concerns labeling. In most of the cases, NEs are not declared in the label. However, transparency in labeling has been highlighted as a key factor influencing consumer trust and acceptance. Furthermore, consumer perceptions around the use of these compounds may vary depending on their knowledge of the benefits of bioactive compounds and their willingness to accept changes in texture or flavor if they are perceived as healthy. While many consumers value health benefits, skepticism toward nanotechnology and concerns about potential alterations in taste or texture may hinder market adoption. Therefore, clear labeling and education on the benefits of these technologies are essential to improve acceptance [197,198].

## 6. Conclusions

The body of evidence examined in this review indicates that the incorporation of NEs into meat products (particularly O/W emulsion matrices, such as patties and sausages) offers a useful technological route to improve product quality, safety, and stability, leading to a shelf-life extension. The reduced DS and extended interfacial area of NEs promote the effective incorporation of lipophilic bioactive compounds, with multiple studies demonstrating improved delivery and bioavailability of essential oils, polyphenols, and omega-3 fatty acids. Moreover, NEs facilitate the use of natural antioxidants and antimicrobials, presenting a viable alternative to synthetic preservatives and aligning with the growing consumer demand for clean-label and health-promoting food products. Studies have shown that incorporating bioactive compounds in NEs leads to shelf-life extension (up to 45 days, depending on dose and formulation characteristics) by reducing microbial growth and increasing oxidative stability and physicochemical properties with lower doses than non-encapsulated forms. Beyond improving shelf-life and microbial safety, NEs have been demonstrated to influence various physicochemical properties, including pH, color, and texture, which are critical to maintaining product integrity and consumer appeal during storage. However, although the physicochemical and antimicrobial advantages of NEs are well documented in meat products, there is a lack of specific information regarding sausages and meat patties. In addition, there is still a significant gap in understanding how the incorporation of bioactive compounds in NEs affects the sensory attributes in these meat products (e.g., flavor, aroma, and mouthfeel) when incorporated. Considering consumer acceptance is a key determinant of commercial success, more comprehensive studies focusing on sensory evaluation are essential. These studies should aim to optimize NE formulations to ensure that functional benefits do not compromise sensory quality. In conclusion, NEs hold immense potential in modern meat processing, but their successful integration into commercial products will depend on striking a balance between functional efficacy and sensory appeal through targeted research and product development.

## Figures and Tables

**Table 1:** Comparison of nanoemulsion preparation techniques [21,33].

Method	Conditions	DS (nm)	Advantages	Limitations	PDI	EE (%)
**HEM**						
HPH	3.45–34.45 MPa	<500	Scalable, uniform DS, low coalescence rate	Multiple passes; viscosity ratio > 80 between phases increases DS	02–04	75–85
MFH	270 MPa	50–300	Ultra-small droplets and highly stable DS	High cost and time; recoalescence; requires precise control	01–03	90–90
USH	20 kHz	20–200	Low surfactant requirement	Heat generation limits processing; not scalable	02–04	70–85
RSH	3000–60,000 rpm	100–500	Simple, cost-effective	Produces coarse emulsions (coalescence of droplets)	03–05	60–75
**LEM**						
PIT	↓T for O/W ↑T for W/O	50–200	Cost-effective, scalable, good control	Limited to non-ionic surfactants; high quantity of surfactants	02–04	70–80
PIC	T = constant	<60	Precise droplet control	Time-consuming	01–03	75–90
SE	T = constant	30–100	No external energy needed, scalable	Requires large quantities of specific surfactants	02–04	60–80
ME	0.015–0.3 MPa, 0.1–10 μm	50–300	Uniform DS	Low throughput, costly membranes	01–02	70–85
SD/E	300–1000 rpm, 0.1–10 mL/min	100–500	Good for hydrophobic molecules	Solvent residue concerns	02–05	65–80

Abbreviations: Method: HEM—high-energy method; LEM—low-energy method; HPH—high-pressure valve homogenization; MFH—high-pressure microfluidics; USH—ultrasonic homogenization; RSH—rotor–stator homogenization; PIT—phase inversion temperature; PIC—phase inversion composition; SE—spontaneous emulsification; ME—membrane emulsion; SD/E—solvent displacement/evaporation; Others: DS—droplet size; EE—encapsulation efficiency; PDI—polydispersity index; rpm—revolutions per minute; T—temperature; ↓—low; ↑—high.

**Table 3:** Physicochemical properties and formulation parameters of NEs in different meat products.

Active Ingredient	Type of NE	DS (nm)	Emulsifier	Meat Product	Storage Conditions	Results	Ref.
**Physicochemical quality**							
Essential oil	W/O (II)	165.7	Canola oil	Pork patties	nd	Improved texture and sensorial profile	[48]
Rosemary extract	Gelatin, chitosan (II)	257	Gelatin, chitosan	Carbonado chicken	6 days, 4 °C	Improved shelf-life and sensorial profile	[58]
Clove essential oil	2.25% CMC + 0.5% CEO + 0.125% ε-PL (II)	257.7	CMC	Chilled meat	10 days, 4 °C	↑ SL (refrigerated); significant preservation by maintaining pH stability	[64]
Riboflavin	1% NE UV-C induced photocrosslinking (II)	N.S.	Tween 80 and Span 80	Fresh beef	26 days	↑ SL up to 26 days; ↓ meat reddening, lipid oxidation, moisture vapor transmission, and solubility; ↑ tensile strength	[65]
*Rosa canina* L. extract	O/W (II)		Chia seed gum	Burger	90 days, −14 °C	↑ SL, color, and sensorial acceptance	[19]
Polylysine and nisin	NEAC with 1.5% SPI (II)	97.1	SPI and lecithin	Yao meat	45 days, 4 °C	↑ SL from 8 to 16 days; no effect on meat sample moisture	[66]
**Oxidative stability**							
Curcumin	O/W (II)	N.S	Tween 80	Harbin red sausage	6 days, RT	↓ pH; stabilization of L* value, WL, WHC, and a_w_; ↓ lipid oxidation and proteolysis	[67]
*β*-carotene	UAE coarse W/O NE (II)	260	Tween 80: Span 80 1:2	Goat meat sausage	15 days, 4 °C	↓ spoilage and lipid oxidation; ↑ color likeness score	[68]
TEO	O/W (II)	80	Gum Arabic	Bovine meat	30 days, 4 °C	↓ pH, TVB-N, TBARS, and hardness; ↑ SL for 30 days	[69]
Capsaicin	O/W (II)	632	Sunflower oil	Sausages	30 days, 4 °C	↓ TVB-N amount and protein decomposition; ↑ SL for 30 days; ↑ pH, color parameters, TVB-N, TBARS amounts, and textural properties; antioxidant effects	[70]
Tocopherol	Ultrasonicated O/W NE (II)	~500	Tween 80	Fish sausages	16 days, 4 °C	↑ SL; antioxidant effect; stable DS (no significant aggregation or creaming)	[71]
Mentha, pomegranate, thyme, and lemon EOs and chitosan	O/O (II)	100–170	Tween 80	Minced meat	10 days, 4 °C	↑ antioxidant effect; ↑ SL up to 10 days	[72]
Fennel EO and cinnamaldehyde	O/W (II)	295.7	Tween 80	Pork meat patties	10 days, 4 °C	↑ SL by 4 days; ↑ TBARS value and TVB-N content; maintained moisture, flavor, and texture	[73]
**Microbial growth**							
CA	UAE coarse O/W NE (II)	146.1	Tween 80	Sausages	4 weeks, 4 °C	↓ microbiological growth (*S. aureus* MIC 0.25 mg/mL; *E. coli* MIC 0.20 mg/mL; *C. perfringens* MIC 0.25 mg/mL) due to interfacial cross-linking with proteins	[74]
Thymol	UAE coarse O/W NE (II)	86.39	Tween 80	Sausages	4 weeks, 4 °C	↓ microbiological growth (*S. aureus, E. coli*, and *C. perfringens*); ↑ color quality	[75]
Chitosan	UAE O/W (II)	440	Tween	Chicken sausages	30 days, 4 °C	↑ SL from 20 to 30 days; potential food additive to preserve the quality	[76]
Curcumin	O/W (II)	N.S	Tween 80	Harbin red sausages	6 days, RT	↓ growth of TAB and bacteria abundance; ↑ SL up to 6 days	[67]
Mentha, pomegranate, thyme, and lemon EOs and chitosan	O/O (II)	100–170	Tween 80	Minced meat	10 days, 4 °C	↑ antimicrobial against *E. coli*; ↑ SL up to 10 days	[72]
Carvacrol	NE with 2.5% carvacrol (II)	~21	Lecithin and casein	Minced pork	9 days, 4 °C	↑ SL; ↓ mesophilic, lactic, and psychotropic bacteria; maintained color stability	[18]
Fennel EO and cinnamaldehyde	O/W (II)	295.7	Tween 80	Pork meat patties	10 days, 4 °C	↓ TVC, ↑ SL by 4 days	[73]
Cinnamon EO and rosemary extract	O/W (II)	183.6	Tween 80	Chicken patties	12 days, 4 °C	↓ TVC of *E. coli, S. subtillis*, and *S. aureus*; ↑ SL by 4 days; ↓ TVB-N, moisture, and TBARS values	[77]
Lemon EO	O/W (II)	500	Sodium caseinate	Pork patties	30 days, 4 °C	↑ quality and microbiological status; ↓ TVB-N levels	[78]
Nutmeg and ginger EOs	O/W (II)	129	Tween 80	Beef patties	90 days, −18 °C	↑ quality and SL by 45 days; ↓ concentrations of HCAs and PAHs	[79]
Resveratrol + Clove EO	NE-based basil seed gum (BSG) edible film (ST)	242.1–770.4	Tween 80, Basil seed gum	Minced camel meat	20 days, 4 °C	↑ oxidative stability; ↑ sensory acceptability	[80]
**Combined effect**							
Chitosan–thymol EOs	O/W (II)	N.S	Tween 20	Pork meat	12 days, 4 °C	Antimicrobial against *Pseudomonas*, *Lactococcus*, and *Acinetobacter*; ↑ SL; ↓ TVB-N, pH, and TBAR values; better protection against color degradation	[81]
Chitosan–thyme EOs							
Oregano, cinnamon, lemon, cardamom, and pepper EOs	O/W (II)	2–47	Tween 80	Mortadella	20 days, 14 °C	↑ SL to 20 days; effective against *Clostridium sporogenes*; no changes in technological properties; unsatisfactory sensory effects	[82]
Xoconostle	W/O (II)	N.S	N.S	Sausages	60 days, 4 °C	↑ hardness, chewiness, antioxidant capacity; ↓ lipid oxidation	[83]
Thymol	W/O (II)	86.39	Lipids	Sausages	4 weeks, 4 °C	↑ redness, antioxidant capacity, and color stability	[84]
TEO	O/W (II)	80	Gum Arabic	Bovine meat	30 days, 4 °C	↓ microbiological growth; ↑ SL for 30 days	[69]
TMO (1%, *w*/*v*)	CSCNC-AS aerogels (mass ratio of 1:5) (II)	78.65	Tween 80	Chilled meat	12 days, 4 °C	↓ microbiological growth; ↑ SL for 8 days; stable structure at various T; sustained release of TMO; ↑ thermal stability and water absorption	[85]
Curcumin, GA, and QUE	NE-loaded gelatin composite films (ST)	100	Tween 80 and Span 80	Fresh broiler chicken	17 days, 4 °C	↑ SL: 135 days at 25 °C; ↓ ZP (−28.1 mV, PDI: 0.328) due to polyphenol encapsulation	[86]
Thymol, γ-terpinene, and ρ-cymene	ONE + 1% AEO and ONE + 2% AEO (II)	181	Tween 80 (3%)	Lamb loins	12 days, 4 °C	↓ microbiological growth; ↑ stability and SL from 8 to 12 days; antioxidant effect	[60]
SKEO	Chitosan-based coating (ST)	93	Triton	Lamb meat	20 days, 4 °C	↓ microbiological growth; ↑ SL (refrigerated); maintained quality	[87]
Origanum vulgare	PIT NE 5 g EO/100 g (II)	35	Cremophor RH40 and Span 80	Chicken pâté	8 days, 4 °C	↓ microbiological growth; stable for 5 g EO/100 g; physicochemical properties not altered	[17]
Clove oil and crodamol	NE (ultrasonic emulsification) (II)	135	Tween 80	N.S.	60 days, 4 °C	↑ antimicrobial and antioxidant potential	[59]
EOS	Optimized NE (via RSM and MF) (II)	38.11	Tween 80 and Span 80	Fresh meat	30 days, 4 °C	↑ SL from 5 to 30 days at 4 °C and 30 °C; high encapsulation efficiency (83.8%); ζ potential: 37.8 mV	[88]
OEO and resveratrol	PEC edible coating under HOMAP (ST)	50	Tween 80 and ethanol	Fresh pork loin	15 days, 4 °C	↑ SL by minimizing pH changes and ↓ lipid and protein oxidation; ↓ microbial growth; ↑ preservation function and stability of the coating system	[61]
VD3	NE with 1.54% PP, 9.12% SO, and 0.4% salt (II)	485	PP, SO, and salt	Beef	N.S.	↑ stability after cooking; ζ potential and VD3 retention of −37.76 mV and 55.1%	[89]
Limonene, *β*-Pinene, and Terpinene	Nano-CS-CLEO 2% and 4% (II)	N.S.	Tween 80	Beef	16 days, 4 °C	↓ microbiological growth; ↑ SL; ↓ TBARS, ↑ antioxidant effect and organoleptic retention	[90]
Rosemary extract	NE-based edible gelatin-chitosan coating (ST)	257	ε-poly-L-lysine	Carbonado chicken	16 days, 4 °C	↓ pH changes at 4 °C for 16 days; sustained release of the active compound on the meat surface	[64]
*Zingiber officinale* EO (6% wt)	NE-based edible coating (ST)	57	Sodium caseinate	Fresh chicken	12 days, 4 °C	↑ SL; ↓ total aerobic psychrophilic bacteria and yeasts; ↓color and cooking loss changes	[63]
Geraniol and linalool	NE with 6 g of MCT oil (II)	68.2–174	Tween 80	Meat simulation medium	7 days, 2–5 °C	↑ SL; ↓ *E. coli* and *L. innocua* (~3 log CFU/mL); *P. lundensis* (~1.2 log CFU/mL)	[91]

Abbreviations: II—Internal incorporation; CA—cinnamaldehyde; NE—nanoemulsion; MIC—minimum inhibitory concentration; IZ—inhibition zone; IZ^C^—inhibition zone control; TVB-N—total volatile basic nitrogen; TBARS—thiobarbituric reactive substances; TEO—thyme essential oil; TMO—thymol NE; OPEO—orange peel essential oil; CSCNCs—corn straw cellulose nanocrystals; AS—acetylated starch; Nano-CEO-ε-PLCMC—carboxymethyl chitosan-coated ε-polylysine (ε-PL) NE; CMC—carboxymethyl chitosan; CEO—clove essential oil; GA—gallic acid; QUE—quercetin; ST—surface treatment; FGNE—food-grade NE; ONE—virgin olive oil NE; AEO—ajowan (*Carum copticum*) essential oil; NEAC—NE-based active coating; SPI—soy protein isolate; SKEO—*Satureja khuzestanica* essential oil; PIT—phase inversion temperature; SL—shelf-life; EOs—essential oils; RSM—response surface methodology; E—emulsion; OEO—oregano essential oil; PEC—NE-loaded pectin; HOMAP—high-oxygen modified atmosphere packaging; VD2—vitamin D3; PP—pea protein; SO—safflower oil; CLEO—citrus limon essential oil; Nano-CS—coated with nano-chitosan; NZEOC—coated with corn starch solution containing 1% (*w*/*v*) NE of *Zataria multiflora* essential oil fortified with cinnamaldehyde; *E. coli—Escherichia coli*; *L. innocua—Listeria innocua*; *P. lundensis*—*Pseudomonas lundensis*; MCT—medium-chain triglyceride; RT—room temperature; WL—weight loss; WHC—water-holding capacity; a_w_—water activity; TAB—total aerobic bacteria; EO—essential oil; N.S—not specified; TVC—total viable counts; HCAs—heterocyclic aromatic amines; PAHs—polycyclic aromatic hydrocarbons; ZP—zeta potential; UAE—ultrasonicated; DS—droplet size; ↓—reduced; ↑—increased; nd—not determined.

**Table 4:** Incorporation of bioactive compounds into patties and sausages.

Source	Meat Product	Formulation	Storage	Results	Ref.
**Polyphenols**					
Beer residue extract, chestnuts, leaves, and peanut skin	Spanish salchichón	Powder, 2.0%	-	↓ PO, VC	[102]
Pomegranate, red grape, tomato, and olive pomaces	Lamb patties	Extracts, 0.1%	7 days, 2 °C	↑ PAA; ↓ MG	[103]
Pomegranate peel and bagasse	Chicken patties	Powder, 2%	16 days, 4 °C	↓ PO, LO	[104]
Peanut skin	Chicken patties	Extract, 3%	15 days, 1 °C	↓ LO, a*	[105]
Red pitaya extract	Pork patties	Powder, 0.1%	18 days, 2 °C, MA, fluorescence light	↑ CL, PAA; fat replacement	[106]
Apple peel	Raw beef patties	Edible coating, 3%	10 days, 4 °C	↓MG, LO	[107]
Watermelon rind	Cooked pork patties	Extract, 0.10%	28 days, 4 °C	↑ CA; ↓ MG, LO	[108]
Jaboticaba peel	Beef burgers	Emulsion, 10%	120 days, −18 °C	↑ CA; ↓ PO, LO	[109]
Jaboticaba peel	Meat	Extract, 8 g/L	30 days, 4 °C	↑ CL	[110]
Mango peel	Chicken sausages	Powder, 4%	10 days, 4 °C	↓ PO, LO	[111]
Oregano	Sheep sausages	Extract, 6630.98 mg/kg	135 days, −20 °C	↓ VC, LO	[112]
Sea buckthorn, grape seeds, fenugreek seeds, green tea, and Acacia catechu	Pork frankfurters	Extract, 0.30, 0.10, 0.12, 0.03, and 0.10%, respectively	20 days, 4 °C	↓ LO	[113]
Acacia nilotica seeds	Chicken patties	Extract, 150 mg/100 mL	15 days, 4 °C	↓ MG, PAA	[114]
Blue pea flower petal	Cooked pork patties	Extract, 0.08–0.16%	12 days, 4 °C	↓ VC, LO	[115]
Bee pollen	Pork sausages	Extract, 0.02%	30 days, 4 °C	↓ PO, LO	[116]
Propolis	Beef and pork patties	Extract, 2%	9 days, 4 °C	↑ CL; ↓ PO, LO	[117]
Mastic leaves and fruit	Pork sausages	Extract, 300 ppm	21 days, 4 °C	↓ MG, LO	[118]
Rose	Fermented sausages	Extract, 0.3%	16 days, 10 °C	↓ MG, LO, BAM	[119]
Cherry powder extract	Pork patties	Powder, 20 ppm	8 days, 4 °C, PP	↑ CL, CA; ↓ LO	[120]
Citrus, rosemary, and acerola by-products	Spanish chorizo	Powder, 0.3%	50 days, 4 °C	↓ MG, LO	[121]
Oak wood	Pork patties	Extract, 1%	8 days, 4 °C, MA	↑ CL, CA; ↓ MG, LO, VC	[122]
Black and green tea	Uncured pork sausages	Extract, 0.30 and 0.05%	5 days, 4 °C	↓ PO, LO	[123]
Rosemary	Beef sausages	Extract, 25 ppm	25 days, 4 °C	↑ PAA	[124]
Mint	Beef sausages	Extract, 62 ppm	25 days, 4 °C	↑ PAA, AM	[124]
Hull, bur, and leaf chestnut	Beef patties	Extract, 1000 ppm	18 days, 2 °C	↓ LO	[125]
**Hydroxycinnamic acids, tyrosols**					
Pitanga leaves	Pork burgers	Powder, 250 ppm	18 days, 2 °C, MA, polystyrene film	↑ a*; ↓ MG, LO	[126]
Pitanga leaves	Lamb burgers	Powder, 250 ppm	2 °C, MA	↑ CL; ↓ PO, LO	[127]
Guarana seeds	Lamb burgers	Emulsion, 250 ppm	18 days, 2 °C, PE	↑ CL; ↓ PO, LO	[127]
**Anthocyanidins**					
Plum peel and pulp	Breast chicken patties	Powder, 2%	10 days, 4 °C PE, dark	↑ CL, a*; ↓ LO	[128]
Açaí extract	Pork patties	Powder, 250 ppm	10 days, 2 °C, PE, dark	↑ CL, a*; ↓ LO	[129]
Jaboticaba residue	Fresh sausages	Powder, 2%	15 days, 1 °C, aerobic conditions, dark	↓ LO, CA (2%)	[130]
Colombian berry	Pork patties	Extract, 250–750 ppm	10 days, 4 °C, MA	↑ CL, a*; ↓ LO	[131]
Jaboticaba fruits	Mortadella sausages	Powder, 2%	56 days, 4 °C	↑ T, F	[132]
Jaboticaba residue	Fresh sausages	Powder, 2%	15 days, 1 °C	↓ LO	[130]
**Flavonoids, tannins, terpenoids**					
Thyme by-products	Pork patties	Powder, 0.93%	9 days, 4 °C	↑ CL; ↓ PO	[133]
**β-carotene, lycopene**					
Pink guava pulp	Raw pork emulsion	Paste, 10%	18 days, 2 °C, MA	↑ CL; ↓ LO	[134]
Tomato by-products and pink guava	Pork emulsion	Powder, 10%	9 days, 4 °C, aerobic packaging, dark	↑ CL; ↓ LO	[135]
Pitaya leaves	Pork patties	Powder, 100–1000 ppm	9 days, 4 °C, aerobic packaging	↓ PO	[136]
**Resveratrol**					
Grape seed	Beef sausages	Powder, 300 ppm	4 months −18 °C, PVC	↓ LO	[137]
**Stilbenes**					
Peanut kernels	Pork sausages	Extract, 0.01%	8 days, 4 and 25 °C	↓ MG, LO	[138]
**Catechins, epicatechins**					
Guarana seed	Pork patties	Powder, 250 ppm	18 days, 2 °C, MA	↑ CL; ↓ PO, LO	[139]

Abbreviations: MA—modified atmosphere; PP—propylene bags; PE—polyethylene film; ↑—enhance; ↓—reduce; PO—protein oxidation; VC—volatile compounds; PAA—product antioxidant activity; MG—microbiological growth; LO—lipid oxidation; CL—color stability; CA—consumer acceptance; BAM—biogenic amines formation; a*—improve redness of the color; T—texture; F—flavor.

## Data Availability

No new data were created or analyzed in this study. Data sharing is not applicable to this article.

## Abbreviations

The following abbreviations are used in this manuscript:

**Generic**	
SFA	Saturated fatty acid
NEs	Nanoemulsions
O/W	Oil-in-water
HEM	High-energy method
LEM	Low-energy method
PIT	Phase inversion temperature
SE	Spontaneous emulsification
HPH	High-pressure valve homogenization
MFH	High-pressure microfluidics
USH	Ultrasonic homogenization
RSH	Rotor–stator homogenization
PIC	Phase inversion composition
SD/E	Solvent displacement/evaporation
DS	Droplet size
ZP	Zeta potential
IT	Interfacial tension
W/O	Water-in-oil
DO	Digestible oils
IO	Indigestible oils
IF	Interfacial film
GRAS	Generally regarded as safe
PUFAs	Polyunsaturated fatty acids
TVB-N	Total volatile basic-nitrogen
TBARS	Thiobarbituric acid reactive substances
MIC	Minimum inhibitory concentration
PV	Peroxide value
MBC	Minimum bactericidal concentration
PEFs	Pulse electric fields
**Compounds**	
Fe	Iron
Cu	Copper
Zn	Zinc
Mn	Manganese
MCTs	Medium-chain triglycerides
LCTs	Long-chain triglycerides
BHT	Butyl-hydroxy-toluene
BHA	Butyl-hydroxy-anisole
TBH-Q	Tert-butyl-hydroquinone
PG	Propyl-gallate
ALA	Alpha-linolenic acid
Se	Selenium
RES	Resveratrol
CEO	Clove essential oil
